# Identification and validation of lactate metabolism-related genes in oxygen-induced retinopathy

**DOI:** 10.1038/s41598-023-40492-z

**Published:** 2023-08-16

**Authors:** Jiawei Xu, Yunpeng Zhang, Rong Gan, Zhuoqi Liu, Yan Deng

**Affiliations:** 1 Department of Ophthalmology, The Second Affiliated Hospital of Nanchang University, Nanchang, Jiangxi China; 2 The Second Clinical Medical College of Nanchang University, The Second Affiliated Hospital of Nanchang University, Nanchang, People’s Republic of China; 3 Department of Biochemistry and Molecular Biology, School of Basic Medical Sciences, Nanchang University, Nanchang, People’s Republic of China

**Keywords:** Immunology, Molecular biology, Pathogenesis

## Abstract

Retinopathy of Prematurity (ROP) is a multifactorial disease characterized by abnormal retinal vascular growth in premature infants, which is one of the leading causes of childhood blindness. Lactic acid metabolism may play an imperative role in the development of ROP, but there are still few relevant studies. Our team use a dataset GSE158799 contained 284 genes in 3 P17_OIR mice and 3 P30_OIR mice to identify 41 potentially differentially expressed lactate metabolism-related genes (LMRGs) related to ROP. Then through bioinformatics analysis, we strive to reveal the interaction, the enriched pathways and the immune cell infiltration among these LMRGs, and predict their functions and internal mechanisms. These DEGs may regulate lactate metabolism, leading to the changes of metabolism and immunity, thereby inducing the development of ROP. Our results will expand our understanding of the intrinsic mechanism of ROP and may be helpful for the directions for treatment of ROP in the future.

## Introduction

Retinopathy of Prematurity (ROP) is a multifactorial disease characterized by abnormal retinal vascular growth in premature infants, which is one of the leading causes of childhood blindness^1^. The incidence of ROP is about 60% in very low birth weight (VLBW) infants^2^. The major risk factors of ROP include: low birthweight (< 1500 g), gestational age (< 32 weeks), and extended supplemental oxygen^3^. The pathogenesis of ROP is the result of multiple factors intertwined and acting together. Metabolic changes play an important role in the pathogenesis of ROP. Yang et al. have found that in the metabolic profile of ROP, aerobic glycolysis and lipid metabolism showed an excessive activity^4^. However, based on previous research, we know that babies with ROP are also at higher risk of bronchopulmonary dysplasia (BPD), which is associated with increased glycolysis^5^. Besides, Hartnett et al. have pointed that insufficient glucose metabolism in retinal endothelial cells interfered with physiologic retinal vascular development (PRVD), leading to formation of neovascularization in ROP^6^.

Unlike most other tissues in the body, the purpose of glycolytic pyruvate in the retina is to produce lactate^7,8^. This rapid conversion of glucose to lactic acid is called aerobic glycolysis^9^. Lactic acid is essential for a variety of cell physiological functions and plays a regulatory role in different aspects like energy metabolism and signal transduction^10^. Lactate metabolism is linked to many retinal diseases. For instance, the occurrence of glaucoma and diabetic retinopathy is thought to be related to disturbance of lactate homeostasis^11^. It is reported that some signal pathways can promote ROP through influencing lactate metabolism. Tuten et al. have demonstrated that lactic acid can be used as a marker of poor prognosis in VLBW infants^12^. Also, a study by Singh has showed that the lactate level gradient in the retina is dependent on Hypoxia-inducible factor (HIF), and a HIF-dependent mitochondrial metabolic pathway has been shown to underlie neovascularization^13^. However, the influences of lactate metabolism in ROP still need further exploration. The search for ROP-related LMRGs will provide us with potential biomarkers for ROP and provide a new direction for further exploration of the intrinsic mechanisms and better therapies of ROP in the future.

Binet et al. established an oxygen-induced retinopathy (OIR) mouse model, performed a high throughput sequencing and built a dataset GSE158799. Their research analyzes activated cellular mechanisms of pathological vascular remodeling and regression in retinopathy^14^. The OIR model can be used as a proxy for proliferative retinopathy in humans like ROP. Of note, the prognosis of ROP varies with the clinical stage. Clinically, early stage of ROP has a certain tendency of self-healing. However, for this, we still understand poorly the pathogenesis and the factors determined clinical outcome of ROP. In this study, we selected oxygen-induced retinopathy (OIR) mouse model data at P30 (retinal revascularization) versus P17 (maximal pathological neovascularization). Non-severe ROP has a certain tendency of self-healing, but the specific mechanism is not clear. The significance of this data is that it reflects the formation and regression of pathological neovascularization, which may provide an explanation for this self-healing tendency. We wanted to understand which factors work in this process, and we focused on lactate metabolism. Therefore, we reanalyze this dataset from metabolic and immune perspective and explored the differential expression of genes related to lactate metabolism and various immune cells in ROP. Then, we performed protein–protein interactions (PPI), correlation analysis, gene-ontology (GO) enrichment analysis and Kyoto Encyclopedia of Genes and Genomes (KEGG) pathway enrichment analysis, Gene Set Enrichment Analysis (GSEA), and assessment of immune cell infiltration to differentially expressed LMRGs. Finally, we verified the RNA expression of key DEGs between ROP mice and normal mice by qRT-PCR.We hope that our research can provide new ideas for the internal mechanism treatment of ROP in the future.

## Materials and methods

### Acquisition of lactate metabolism-related genes and RNA-Seq data

The set of 284 genes related to lactate metabolism were retrieved from the Molecular Signatures database (https://www.gsea-msigdb.org/gsea/msigdb). The RNA-Sequencing dataset of GSE158799, on the GPL18635 platform (Ion Torrent Proton), was downloaded from Gene Expression Omnibus (GEO) of the National Center for Biotechnology Information (NCBI) (https://www.ncbi.nlm.nih.gov/geo/). In GSE158799, a total of 6 gene expression data of mouse retina tissue lysates samples were selected, including two groups of OIR PN 17 (3 replicates) and OIR PN 30 (3 replicates). In order to maintain environmental consistency, it is recommended to use litter born mice as controls. The mice undergoing OIR (oxygen-induced retinopathy) were exposed to 75% oxygen from postnatal day 7 to postnatal day 12, where ambient humidity and temperature remain constant^15^. In day 12, they took the mice out of the oxygen chamber, returned them to air room condition (21% oxygen). The hypoxic-driven retinal neovascularization began to develop around day 14^16^. And they were then euthanized on postnatal day 17 and postnatal day 30 to collect retina tissue, respectively. In the meanwhile, the control group were kept in room air (21% oxygen) for the entire duration of the experiment. Throughout the study, all experimental animals were housed in animal facilities with a 12-h light–dark cycle, free access, and normal food and water.

### Identification of differentially expressed genes (DEGs)

Raw data was normalized using the “limma” package in R software (version 4.1.0) and then was annotated by “AnnoProbe” package prior to differential analysis.The P-value and fold change were used to screen the differentially expressed genes (DEGs) from mice retina tissue datasets, with 4294 genes satisfying |log2 fold change (FC)|≥ 0.585 and adjusted P-value < 0.05. The list of DEGs was then intersected with the lactate metabolism-related genes^14,17^. Volcano plot and Heatmap were drawn for further analysis, with R package pheatmap(v1.0.12)^18^ and ggplot2(v3.4.2)^19^.

### PPI analysis and correlation analysis

To explore the potential molecular mechanisms, the PPI analysis of differential genes was performed with STRING database (https://cn.string-db.org/). PPI network was plotted using “ggraph” packages of R software. Gene expression correlation analysis was performed using the Spearman correlation coefficient. Correlation matrix was visualized with the “corrplot” package of R software.

### GO and KEGG pathway enrichment analysis and gene set enrichment analysis

To analyze the potential biological functions of these differentially expressed lactate metabolism-related genes, Gene Ontology (GO) and Kyoto Encyclopedia of Genes and Genomes (KEGG) Pathway analysis were performed through the “clusterProfiler” and “plot” packages of R software. Gene set enrichment analysis (GSEA) was conducted with defined gene sets obtained from the Molecular Signatures database. Significant enrichment was set as adj. p-value < 0.05 and false discovery rate (FDR) < 25%, followed by visualization using the “ggplot2” package of R software.

### Assessment of immune cell infiltration

The types and proportions of infiltrating immune cells in RNA-seq samples were predicted using the “CIBERSORT” package, followed by visualization using “corrplot” package of R software. Box plot were drawn to demonstrate the differential expression levels of the 25 immune infiltrating cells between PN30 and PN17.

### Mouse model of OIR

Mouse model of OIR C57Bl/6 J mice were purchased from.

Hunan SJA Laboratory Animal Co., Ltd. The study was approved by Ethics Committee of Department of Experimental Animals, Medical College of Nanchang University, all methods were carried out in accordance with relevant guidelines and regulations. This study was carried out in compliance with the ARRIVE guidelines. Newborn mice along with their mothers were exposed to 75% oxygen for 5 days from postnatal day PN7 to PN12, and then returned from PN12 to PN17 to normal conditions (21% oxygen). The eyeballs of 17-day-old mice were removed after isoflurane inhalation anesthesia for use in experiments.

### mRNA isolation and quantitative real-time (qRT-)PCR

Total RNA was isolated from retinal tissue using TRIzol reagent (Invitrogen,Carlsbad,CA, USA) according to the manufacturer’s protocol, quantified with a Nanodrop (NanoDrop Technologies,Wilmington),and reverse transcribed into complementary deoxyribonucleic(cDNA) using HiScript II Q RT SuperMix for qPCR(Vazyme Biotechnology,Nanjing,China).StepOnePlus™ Real-Time PCR System(Applied Biosystems,Foster City,CA,USA) with SYBR green quantification was used for Real-time quantitative PCR. Target gene expression levels relative to the level of the internal control β-actin were calculated with the 2 − ΔΔCt method. The primer sequences used in this study were as follows: GAA: 5′-GGGCCTGCACCCTTATCTC-3′(forward),5′-GGTCGGTACGTCTTCCACAG-3′ (reverse);SURF1:5′-ACTTGGCAGGTCCAACGTC-3′(forward),5′- GATGGGCTCAGCCATGACTC-3′(reverse);

HPDL:5′-CCACTAGGGTGAGAGATGCAC-3′(forward),5′- AGCAACGTAAGGCTTAGATTACC-3′(reverse);SLC25A10:5′-GGCTGTATTCCCCTCCATCG-3′(forward),5′- CCAGTTGGTAACAATGCCATGT -3′(reverse);SLC25A4:5′-GGCTGTATTCCCCTCCATCG-3′(forward),5′- CCAGTTGGTAACAATGCCATGT-3′(reverse);POMGNT2:5′-GGCTGTATTCCCCTCCATCG-3′(forward),5′- CCAGTTGGTAACAATGCCATGT -3′(reverse);β-actin:5′-GGCTGTATTCCCCTCCATCG-3′(forward),5′- CCAGTTGGTAACAATGCCATGT -3′ (reverse).

### Statistical Analysis

The statistical analyses were performed using R software (version 4.1.0). Gene expression levels of our clinical samples were compared using Student’s t-test. P < 0.05 was considered statistically significant.

## Results

### Identification of differentially expressed lactate metabolism-related genes (LMRGs)

**Figure 1:**
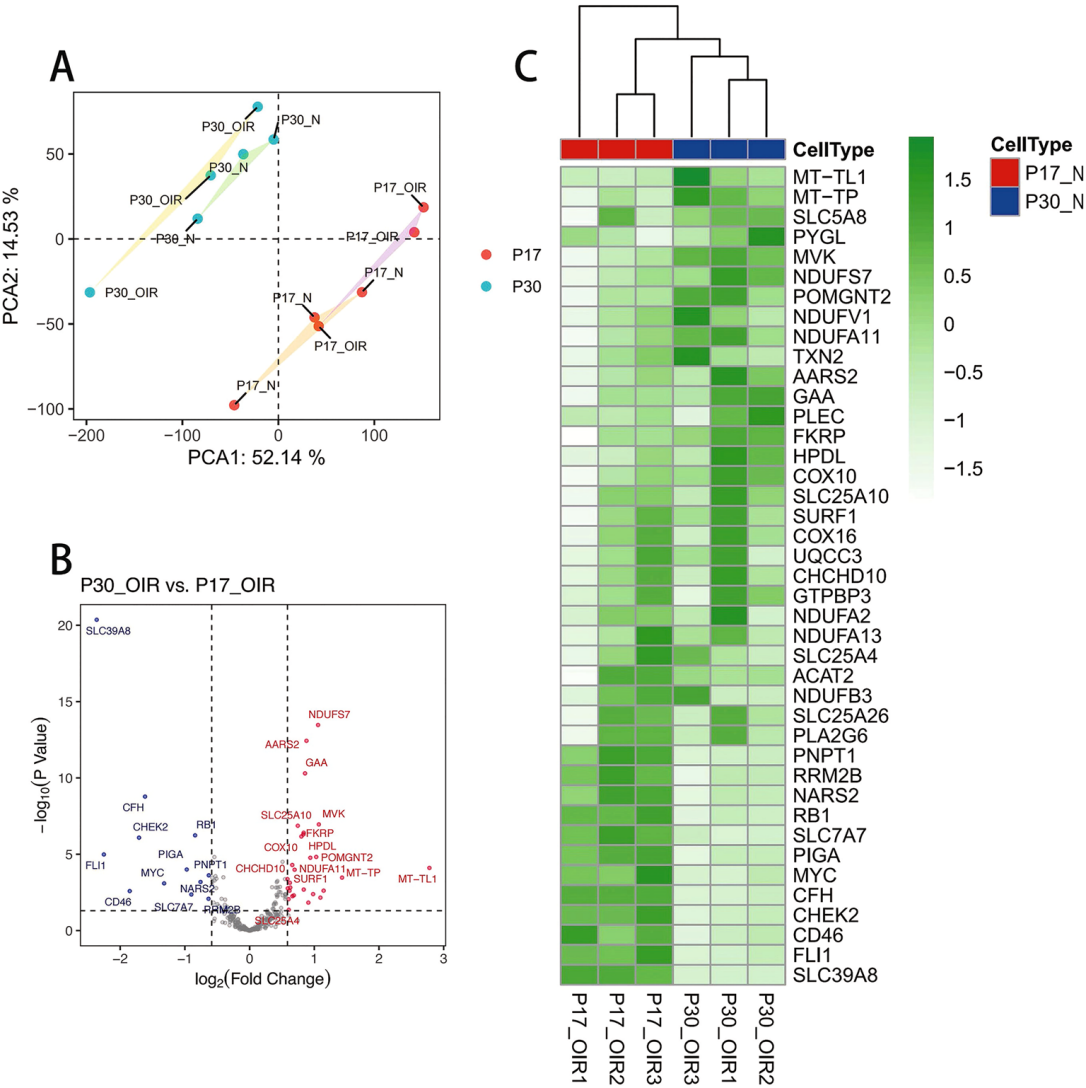
Differentially expressed lactate metabolism-related genes in ROP and control samples. (**A**) Principal component analysis for GSE158799. (**B**) Volcano plot of the differentially expressed lactate metabolism-related genes. The red dots represent the significantly up-regulated genes and the blue dots indicate the significantly down-regulated genes. (**C**) Heatmap of the 41 differentially expressed lactate metabolism-related genes in ROP and control samples.

The two-dimensional PCA clustering map showed the overall characteristics difference of P17_OIR and P30_OIR samples in expression profiling (Fig. 1A), displaying distinct group-bias clustering. Subsequently, we obtained 41 differentially expressed lactate metabolism-related genes (LMRGs) by intersecting 284 genes related to lactate metabolism with the DEGs in 3 P17_OIR mice and 3 P30_OIR mice (Supplementary). For further comparison and visualization, the volcano plot presented the main DEGs in this dataset, as shown in Fig. 1B, C is a heatmap showing the expression levels of the above 41 genes. The up-regulated genes included NDUFS7, AARS2, GAA, MVK, SLC25A10, HPDL, COX10, FLI1, POMGNT2, NDUFA11, CHCHD10, MT-TL1, SURF1, MT-TP and SLC25A4, while down-regulated genes included SLC39A8, CFH, FKRP, RB1, CHEK2, PIGA, PNPT1, NARS2, MYC, CD46, SLC7A7 and RRM2B (ranked by adj. p value).

### PPI network analysis and correlation analysis

**Figure 2:**
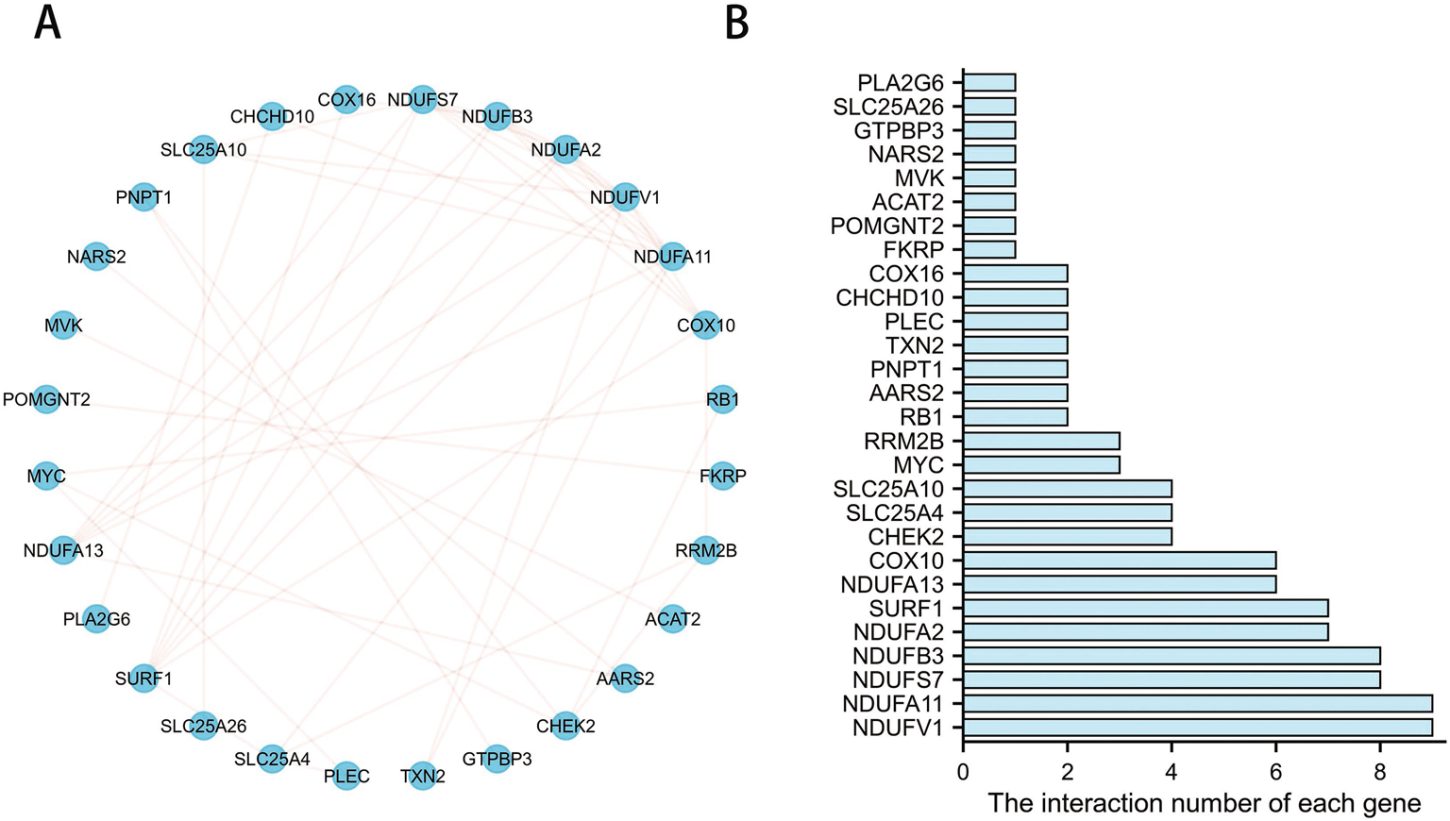
Protein–protein interactions (PPI) analysis the 41 differentially expressed lactate metabolism-related genes. (**A**) The PPI among 41 differentially expressed lactate metabolism-related genes. (**B**) The interaction number of each differentially expressed lactate metabolism-related gene.

**Figure 3:**
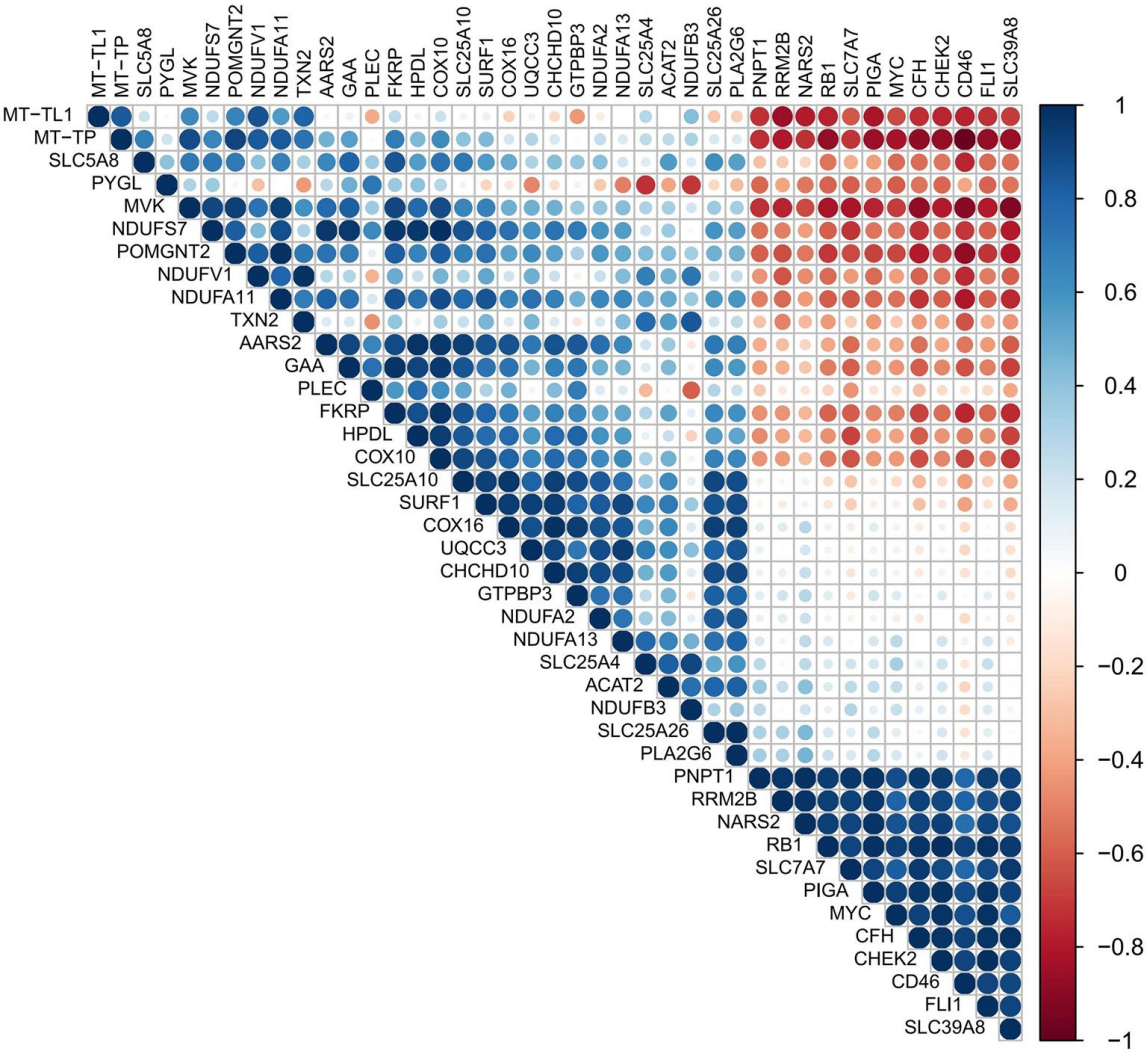
Spearman correlation analysis of the 41 differentially expressed lactate metabolism-related genes. To explore the expression correlation of these lactate metabolism-related genes, correlation analysis was performed. The red dots represent the significantly negative correlation and the blue dots indicate the significantly positive correlation.

The circle chart of PPI network contained 28 nodes in Fig. 2A, demonstrating the interactions between functional proteins of those different expression LMRGs, and the interaction number of each other was presented in Fig. 2B. Correlation heatmap was drawn to show the expression correlation of the 41 differentially expressed LMRGs in GSE158799 dataset (Fig. 3).

### GO function and KEGG pathway enrichment analysis

**Figure 4:**
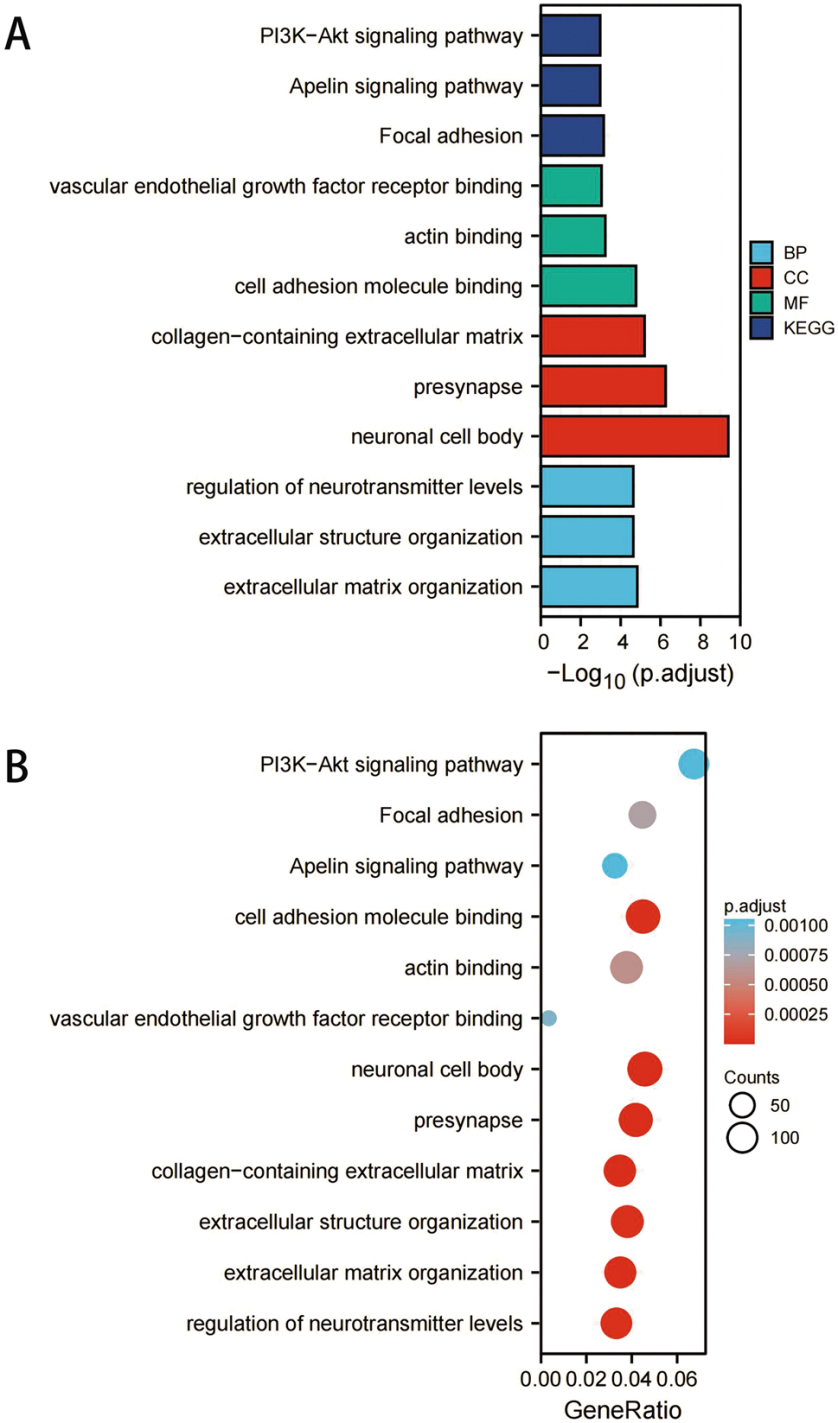
Gene Ontology (GO) enrichment analysis of 41 differentially expressed lactate metabolism-related genes. (**A**) and (**B**) plot of enriched GO terms. The size of the points represents the number of differential genes in the pathway, and the color of the point represents the P value of the hypergeometric test. Abbreviations: BP, biological process; CC, cellular component; MF, molecular function; KEGG, Kyoto Encyclopedia of Genes and Genomes.

**Figure 5:**
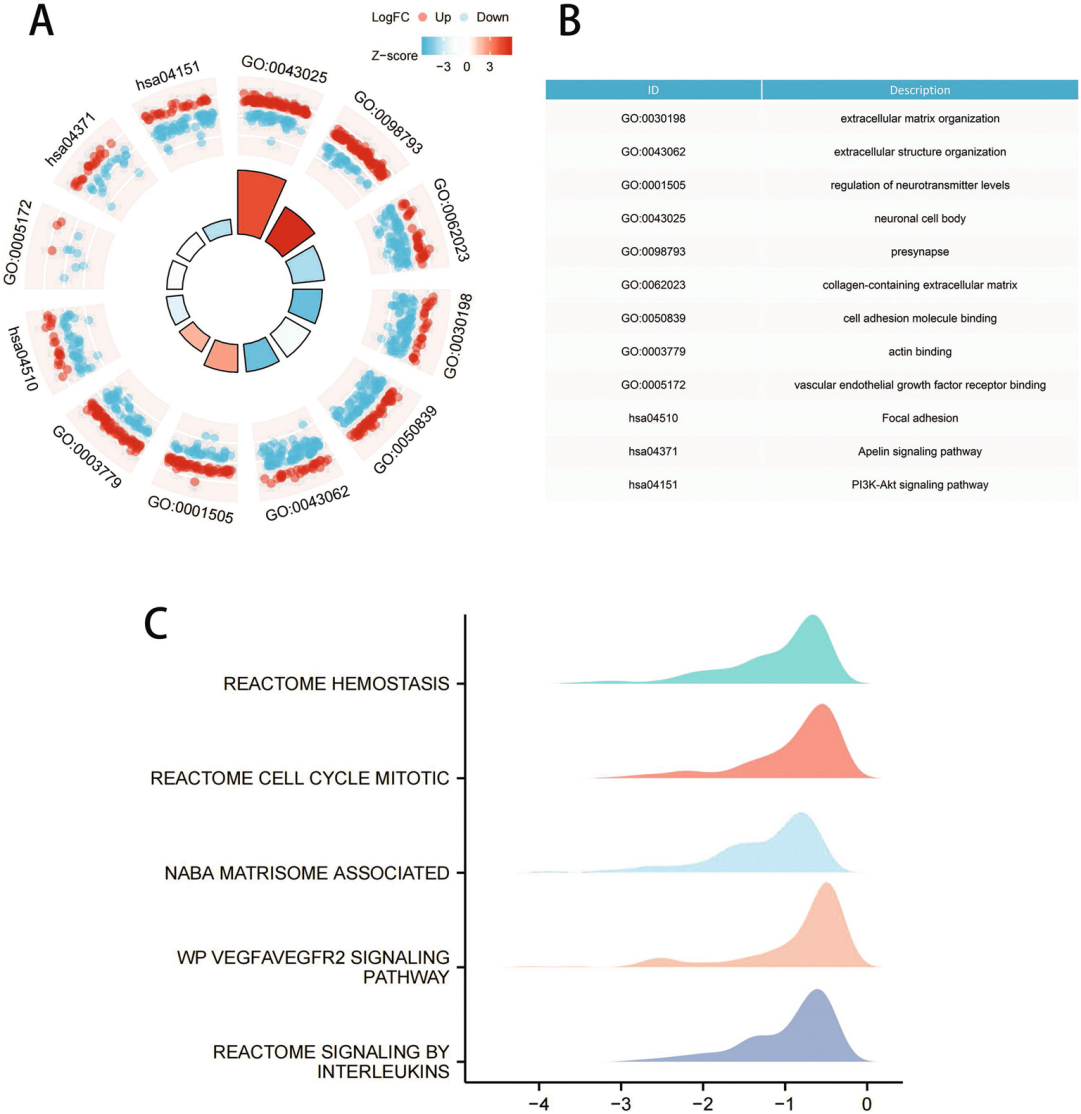
Kyoto Encyclopedia of Genes and Genomes (KEGG) analysis and Gene Set Enrichment Analysis (GSEA) of 41 differentially expressed lactate metabolism-related genes. In the KEGG analysis, the PI3K-Akt signaling pathway was the most enriched pathway by 89 genes. Then we use the GSEA analysis to analyze the signaling pathways enrichment in different groups of a total of 162 gene sets.

In GO analysis, the differentially expressed LMRGs mainly involved in the terms of biological processes including extracellular structure organization (110 genes) and extracellular matrix organization (101 genes). Additionally, several essential molecular functions and cellular components were enriched in, containing neuronal cell body and cell adhesion molecule binding (Fig. 4). In the KEGG pathway analysis, the PI3K-Akt signaling pathway (89 genes) was the most enriched pathway, followed by the Apelin signaling pathway (43 genes) (Fig. 5A,B). These results above identified the biological processes and aberrant signal pathways involved in the progression of ROP.

### Gene set enrichment analysis (GSEA)

A total of 162 gene sets were significantly enriched in GSEA, of which the top gene sets were mainly enriched higher in "reactome hemostasis", "reactome cell cycle mitotic", "naba matrisome associated", "wp vegfavegfr2 signaling pathway" and "reactome signaling by interleukins" (Fig. 5C). Partial results are consistent with GO and KEGG analysis.

### Identification of the expression of screened LMRGs in different kinds of immune cells

**Figure 6:**
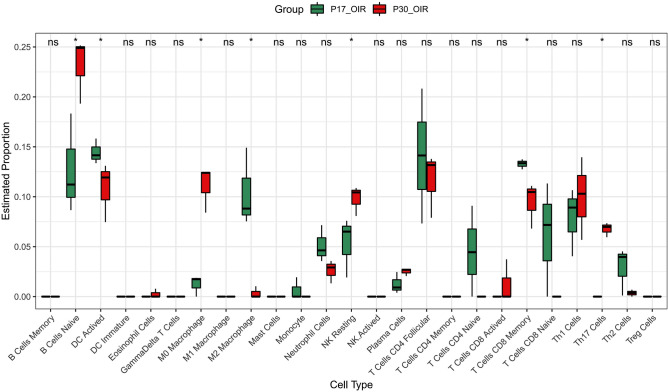
Identification of the expression of screened LMRGs in different kinds of immune cells. The green columns represent the P17_OIR group. The red columns represent the P30_OIR group. We obtained significant differences in seven immune cell infiltration, including B cells Naive, DC Acticed, M0 Macrophage, M2 Macrophage, NK Resting, T Cells CD8 Memory and Th17 Cells.

To evaluate abundance of immune infiltrates and quantify immune cell composition, we analyze the immune cell expression levels among P17_OIR and P30_OIR samples. The analysis showed that among the 25 types of immune cells, there were 7 types of immune cells with significant differences in expression, namely B Cells Naïve, DC Actived, M0 Macrophage, M2 Macrophage, NK Resting, T Cells CD8 Memory and Th17 Cells (Fig. 6). The results demonstrated that the infiltration of M2 macrophages in P30_OIR samples was significantly lower than that in P17_OIR samples and the opposite was true for M0 macrophages, while the infiltration of M1 macrophages had no significant difference between the two groups. The results above suggest that the imbalance of M1/M2 macrophage are essential in the pathophysiological mechanisms of the ROP.

### Validation the differentially expressed LMRGs in OIR group

**Figure 7:**
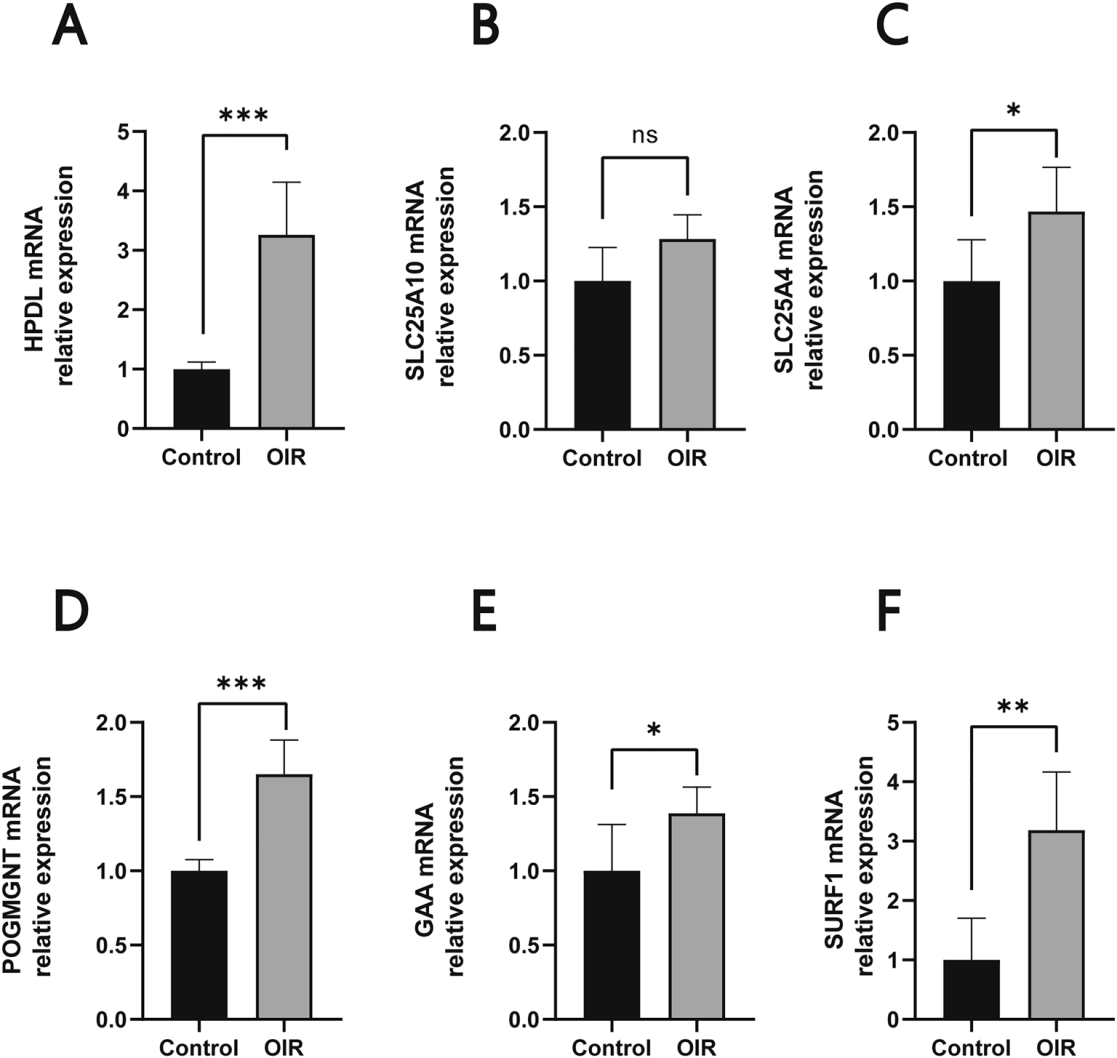
Validation the Differentially Expressed LMRGs in OIR group. Validation of the expression levels of the six hub genes between normal retinal samples (n = 5) and OIR samples (n = 5) by PCR analysis. All of the data are presented as means ± SD. Significant differences were defined by a *p*-value, *P < 0.05, **P < 0.01,***P < 0.001.

To better characterize the expression levels of the six hub genes in normal and OIR tissues, 5 normal retinal samples and 5 OIR samples were collected. As shown in Fig. 7, compared with normal retinal samples, the expression levels of GAA, SLC25A10, SLC25A4, HPDL, POGMGNT and SURF1 were significantly increased in OIR samples (*p* < 0.05).

## Discussion

Retinopathy of prematurity(ROP), the leading cause of childhood blindness worldwide, is a kind of eye disease characterized by progressive abnormal growth of retinal blood vessels and pathological neovascularization in premature infants, which can lead to retinal detachment and blindness in severe cases^20^. The pathogenesis of ROP is divided into two stages mainly^21^. In the first stage, high oxygen environment induces retinal vasoconstriction and decreases vascular endothelial growth factor (VEGF) and insulin growth factor-1 (IGF-1) levels, resulting in blocked retinal vascularization. While in the second stage, retinal ischemia increases the level of HIF-1, which triggers increases of VEGF and IGF-1. This will result in hyperplasia of the retinal blood vessels and increased angiogenesis. If it is not treated in time and correctly, it may eventually lead to traction retinal detachment and visual impairment, even blindness^22^. Recently, more and more studies showed that lactate metabolism may be involved in the occurrence and development of ROP. However, there is little research in this field and further research is needed to better understand. In our study, we use the method of bioinformatics to explore the LMRGs of ROP for the first time. Firstly, we screen out the differentially expressed LMRGs related to ROP from the GSE158799 dataset. After that, we show the association among these genes. And we perform further search for enriched functions and processes in an attempt to reveal their influence on metabolism and immunity in the pathogenesis of ROP.

In this study, we screened 41 potential LMRGs in ROP by bioinformatics analysis (Fig. 1). Some of the ROP-related LMRGs we screened have been previously studied in other retinal diseases. For example, mutations in the mevalonic kinase (MVK) gene were found to affect the occurrence of retinitis pigmentosa (RP) through the mevalonate pathway^23^. Mutations in the SLC39A8 (Zip8) gene and the complement factor H gene (CFH) have been demonstrated to be linked to age-related macular degeneration (AMD)^24,25^. Glutathione (GSH) can act as an important cellular antioxidant in retinal tissue. And SLC25A10 was found to be highly expressed in human retinal pigment epithelium (RPE) cells and retinal layers, which can affect the transport of GSH^26^. Moreover, previous studies have found that friend leukemia integration 1 (FLI1) plays a part in vascular stability during retinal neovascularization^27^. In the future, we will explore more potential ROP-related LMRGs and molecular mechanisms in them.

Some published articles demonstrate that lactate metabolism can influence the progression of ROP. As early as 1976, Gerke et al. have studied the possible role of lactic acid in the formation of retinal poor perfusion neovascularization, and they also pointed that the occurrence of ROP is the result of the joint action of multiple metabolic factors^28^. Tuten et al. have found that lactic acid expression can predict the prognosis of VLBW infants, while low birth weight is one of the most important risk factors for ROP^12^. Besides, Singh et al. has showed that the lactate level gradient in the retina is dependent on HIF, which is widely believed to be associated with neovascularization^13^. We analyzed the potential biological functions of DEGs by GO and KEGG enrichment analysis and found several enriched terms related to lactate metabolism (Fig. 3). For example, Xu et al. have found that PI3K − Akt signaling pathway can induce the glycolytic enzyme lactate dehydrogenase A (LDHA) production, which is the key enzyme in glycolysis. Likewise, the lack of LDHA also inhibit this signaling pathway through reducing production of adenosine triphosphate (ATP) in T cells^29^. Apelin, a group of small peptides, is an endogenous ligand for angiotensin type 1 receptor associated protein. Yuan et al. have demonstrated that apelin-13 can regulate ROS and inhibit glycolysis of macrophages, thereby reducing the release of inflammatory cytokine and improve acute lung injury^30^. Our GSEA analysis also further corroborated the above results.

Recent studies have shown that macrophages play a role in angiogenesis and are involved in retinal and choroidal neovascularization. A clinical study on ROP found that serum MCP-1, macrophage inflammatory protein 1 alpha (MIP-1α) and macrophage inflammatory protein 1 beta (MIP-1β) were significantly elevated in ROP patients. The polarization of the M1 and M2 phenotypes plays an extremely important role in the function of macrophages. An animal experiment by Zhou et al. showed that M2 macrophages enhanced pathological neovascularization in an OIR mode, which are mediated by various cytokines and molecules^31^. Colegio et al. have found experimentally that lactic acid produced by tumor cells can induce the expression of vascular endothelial growth factor (VEGF) and M2-like polarization of tumor-associated macrophages, which it is mechanistically mediated by hypoxia-inducible factor 1α (HIF1α)^32^. It is worth mentioning that the expression of VEGF in tumor-associated macrophages is significantly higher than that of all other cells in the tumor. In 2018, Song et al. demonstrated the role of lactic acid in promoting macrophage-induced neovascularization in the choroid^33^. It is well known that the abnormal changes of VEGF and HIF1α are of great significance in the occurrence of ROP: HIF1α is a key mediator of physiological and pathological angiogenesis through direct regulation of VEGF^34^. Our immune infiltration results showed that the infiltration level of M2 macrophages in P17_OIR group was significantly higher than that in P30_OIR group, while the results of M0 macrophages were just the opposite (Fig. 4). Therefore, we hypothesize that lactic acid-promoted M2 polarization of macrophages play a key role in the regression process of pathological vessels of ROP. And we think that targeting lactate metabolism genes and regulating macrophage polarization may be a new treatment strategy to ROP by promoting the regression of pathological blood vessels and rebuilding normal retinal blood vessels.

We also acknowledge some limitations in our study. First, the number of mice in the GSE158799 dataset is kind of low, which should be considered a limitation. Secondly, the mouse model was used in both the experiment of raw data and our experimental verification, which is a preliminary exploration of the relationship between ROP and lactic acid metabolism. If possible, it is better that we can conduct large-scale studies in ROP patients. Third, we did not verify or explore the underlying mechanisms of these genes in ROP cells, animals and human-focused studies. Therefore, the deep explanation for our results needs to be further explored in the future.

## Conclusion

In conclusion, we identified 41 potential ROP-related LMRGs via bioinformatics analysis. The key genes may affect metabolism and immunity by regulating lactate metabolism, thus leading to the occurrence of ROP. Our results expand the understanding of underlying mechanisms of ROP and it also may be helpful in the treatment of ROP.

## Data Availability

All online data described in this article are available from their web servers and are free for any scientist to use for non-commercial purposes. Further information and source code are available from the corresponding author upon reasonable request.
